# Robust phase-retrieval-based X-ray tomography for morphological assessment of early hepatic echinococcosis infection in rats

**DOI:** 10.1371/journal.pone.0183396

**Published:** 2017-09-08

**Authors:** Huiqiang Liu, Chuanshan Zhang, Xiaoxi Fan, Yingni Duan, Tiqiao Xiao, Guohao Du, Yanan Fu, Haigang Liu, Hao Wen

**Affiliations:** 1 College of Medical Engineering and Technology, Xinjiang Medical University, Urumqi, China; 2 State Key Laboratory Incubation Base of Xinjiang Major Diseases Research, FirstAffiliated Hospital, Xinjiang Medical University, Urumqi, China; 3 SSRF, Shanghai Institute of Applied Physics, Chinese Academy of Sciences, Shanghai, China; Pennsylvania State Hershey College of Medicine, UNITED STATES

## Abstract

Propagation-based phase-contrast computed micro-tomography (PPCT) dominates the non-destructive, three-dimensional inner-structure measurement in synchrotron-based biomedical research due to its simple experimental setup. To quantitatively visualize tiny density variations in soft tissues and organs closely related to early pathological morphology, an experimental study of synchrotron-based X-ray PPCT combined with generalized phase and attenuation duality (PAD) phase retrieval was implemented with the hepatic echinococcosis (HE) infection rat model at different stages. We quantitatively analyzed and evaluated the different pathological characterizations of hepatic echinococcosis during the development of this disease via our PAD-based PPCT and especially provided evidence that hepatic alveolar echinococcosis invades the liver tissue and spreads through blood flow systems with abundant blood supply in the early stage. Additionally, the infiltration of tiny vesicles in HE lesions can be clearly observed by our PAD-PPCT technique due to the striking contrast-to-noise ratio (CNR) and mass density resolution, which cannot be found by the medical imaging techniques, such as magnetic resonance imaging (MRI), computed tomography (CT), and ultrasound, in hospitals. The results demonstrated that our PAD-PPCT technique has a great potential for indicating the subtle structural information of pathological changes in soft biomedical specimens, especially helpful for the research of early micro-morphology of diseases.

## Introduction

Hepatic echinococcosis (HE) arises from the intrahepatic growth of *Echinococcus multilocularis* larvae and generally spreads to other tissue and organs by the infiltration of small vesicles. Similar to a slowly developing malignant tumor, it has a high mortality rate and a high incidence in countries of the northern hemisphere that feature the industry of animal husbandry. The micro-morphological characterization of HE is essential to assessments of disease development, metastases and therapeutic effects at different pathological stages, especially for the prolonged drug therapy of albendazole (ABDZ) inpreventing HE parasite growth [1–3]. A variety of imaging techniques have been employed in the investigation of HE lesion severities in recent decade, including morphological and functional imaging methods. However, an effective early detection of HE is usually hard to achieve based on conventional imaging techniques due to atypical characteristics of HE, and a definitive diagnosis may further rely on serological assessment [4–6]. Therefore, it is necessary to present a new imaging method to assess the morphological HE lesions at an early stage. Although the typical imaging characteristics of HE by ultrasound (US), computed tomography (CT) and magnetic resonance imaging (MRI) have been reported with their own clinical features and advantages [7–12], further three-dimensional, high-contrast, and micron-scale morphological research on *ex vivo* HE pathology is essential and required to assess the HE lesion micro-changes and developing mechanism. With respect to optical or electron microscopy, the resolution can achieve a quasi-nanoscale observation on the basis of micron-level slices and staining that cannot meet the needs of nondestructive three-dimensional (3D) measurement. In our work, we presented the employment of novel synchrotron-based X-ray computed tomography to study the high-resolution 3D characteristic structure and pathological morphology of HE lesions at an early and developed stage.

In many interactions with X-rays and matter, an attenuation effect has been widely and successfully used for medical imaging applications for several decades. Conventional attenuation-based X-ray imaging, due to the penetration power of X-rays, is the central imaging technique for observing internal structures of opaque objects nondestructively [13–16]. However, it is difficult to reveal weakly absorbing structures of an object consisting of low-Z materials, such as soft tissues and organs or light materials, because these structures are almost transparent to hard X-rays. Thus, attenuation-based imaging typically produces poor image contrast for low-Z samples. When X-rays traverse an object, the X-ray undergoes a considerably large phase shift as well, described by the X-ray complex refractive index *n*(*x*, *y*, *z*) = 1 − *δ*(*x*, *y*, *z*) + *iβ*(*x*, *y*, *z*), where the decrement of the refractive index *δ*(*x*, *y*, *z*) is approximately 1000 times higher than the absorption coefficient *β*(*x*, *y*, *z*) for low-Z elements using hard X-ray imaging. Thus, acquisition of the phase contrast, rather than attenuation contrast, is anticipated to provide a much higher image contrast and a lower radiation dose for low-Z biomedical samples. Therefore, X-ray phase sensitive imaging techniques enable us to nondestructively observe inner weak-absorption structures with high phase sensitivity, which is particularly suitable for visualizing and quantifying important information of structures with fine mass density differences in early pathological micro-morphology research or material sciences [17–23]. Currently, hard X-ray propagation-based phase-contrast computed micro-tomography (PPCT) techniques, particularly based on synchrotron radiation facilities, are widely and successfully applied to acquire non-destructive three-dimensional datasets of soft biomedical specimens due to the simpler experimental setup compared to other hard X-ray imaging techniques. The PPCT technique is the most direct method to achieve phase contrast by placing a detector at a distance downstream of an object without any additional X-ray optics required, which renders phase variations visible through translating the phase shift into the accessibly recorded intensity variations. The reconstructed intensity distribution contains the 3D map of the linear attenuation coefficients and the 3D map of Laplacian refraction index decrements of a sample, scaled by the object-detector distance [24–28]. To obtain the quantitative extraction of pure phase maps, phase retrieval algorithms were proposed on the basis of the general X-ray intensity propagation equation or the simplified transport of intensity equation (TIE) [29–36]. There are several studies on different propagation-based phase-contrast micro-tomography methods for biological samples. Their adopted phase retrieval methods of the propagation-based techniques used in these studies were limited in their performance. For example, in these studies, the single-distance phase retrieval method based on the homogeneous material assumption was used. According to this simplifying assumption, the ratio *δ*(*x*, *y*, *z*) / *β*(*x*, *y*, *z*) of the decrement of the refractive index over the absorption coefficient should be assumed to be constant across the specimen volume. To perform phase retrieval with this method, one has to assume *a priori*a value of *δ*(*x*, *y*, *z*) / *β*(*x*, *y*, *z*), which was usually set to the *δ*(*x*, *y*, *z*) / *β*(*x*, *y*, *z*) value of water at the given X-ray energy. However, for the biological soft tissue samples imaged in these studies, the tissues are inhomogeneous; hence, the actual *δ*(*x*, *y*, *z*) / *β*(*x*, *y*, *z*) is not a constant, and it varied by approximately 300% as reported in a study. The breaking down of the constant *δ*(*x*, *y*, *z*) / *β*(*x*, *y*, *z*) assumption in these comparative studies adversely affects the validity of the contrast and density resolution analyses of these studies. Therefore, a single-distance phase retrieval method valid for inhomogeneous low-Z material such as soft tissues is desirable. For better use of the robust phase retrieval tool for pathological micro-morphological research of hepatic *echinococcosis*, a robust single-distance phase retrieval method applicable to inhomogeneous soft tissue samples was presented and demonstrated to be applicable to our HE disease research. In brief, we combined our different propagation-based phase retrieval method, namely, the so-called phase-attenuation duality (PAD) method [37–43], with quantitative measurement of biomedical specimens by using hard X-rays from synchrotron radiation. This PAD method of phase retrieval is based on the fact that for low-Z material such as soft tissues, regardless of homogeneous or inhomogeneous compositions, the electron density completely determines both the decrement of the refractive index and the absorption coefficient for hard X-rays, which will be explained in detail in Section II.

## Materials and methods

### HE specimens

**Fig 1:**
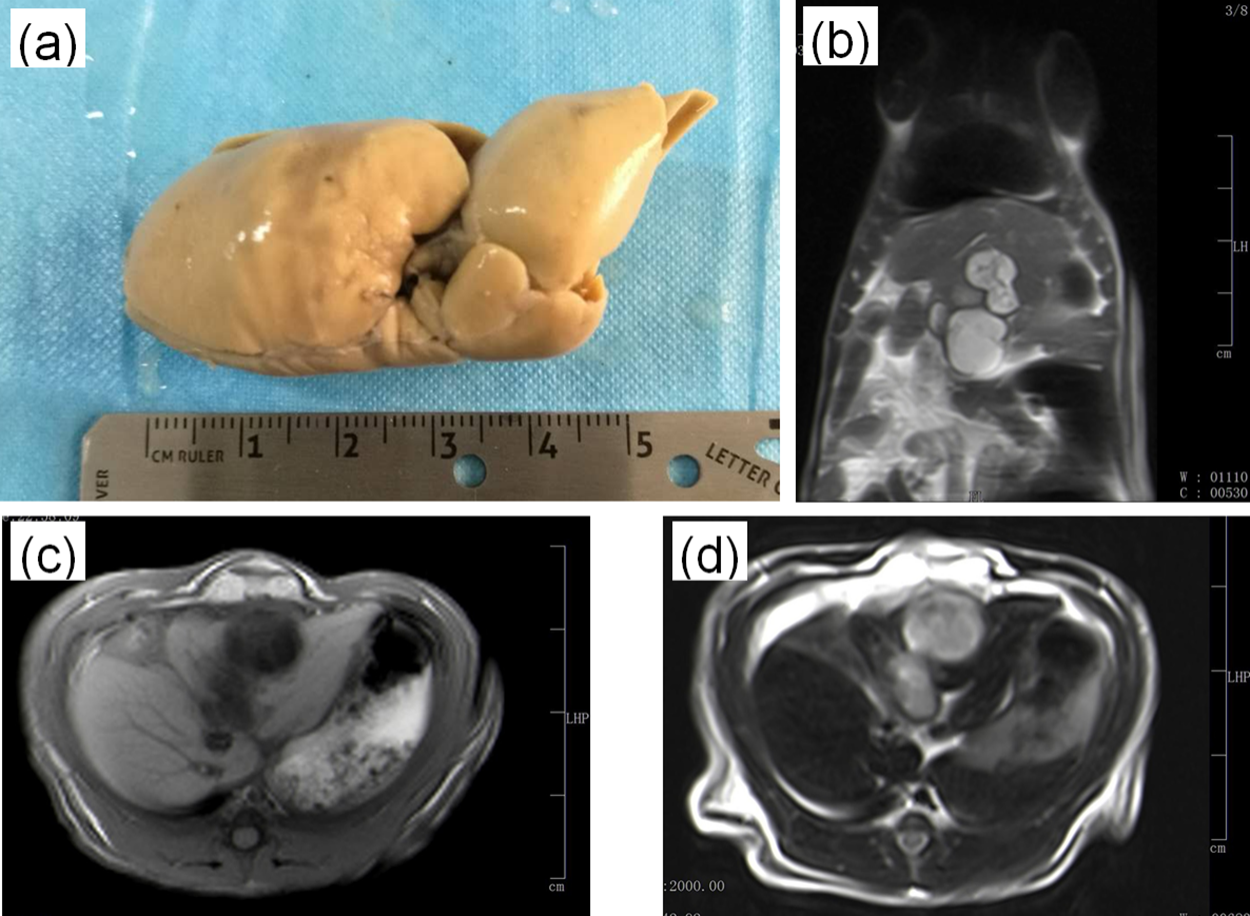
Hepatic echinococcosis (HE) experimental specimen from the two-month disease rat model (a) and the corresponding MRI examination results: (b) Coronal section image; (c) Transverse section image (T1WI); (d) Transverse section image (T2WI). The unit of length of the scalebars in (b-d) is 1cm.

The biomedical specimens used for this study were two groups of HE rat models, totaling 40 SD male rats (200 ± 20g) equally divided into a two-week feeding group and a two-month feeding group after HE inoculation, provided by the breeding center of the First Affiliated Hospital of Xinjiang Medical University (XJMU), China. They were euthanized immediately by cervical dislocation, and the *ex vivo hepatic echinococcosis* HE specimens without staining were kept in formalin solution (10% formalin neutral buffer solution) at room temperature. The operative procedures were carried out by strictly conforming to the guide for the care and use of laboratory animals, and the experimental protocol was approved by the animal experiment ethics committee of the First Affiliated Hospital of XJMU, China. Both HE groups were screened by using an MRI scanner (GE USA), while only the two-month group can be diagnosed as the HE rat models definitively, as shown in Fig 1. The two-week group cannot be confirmed due to the limited resolution of MRI for the early HE lesions. A process of serial graded dehydration with ethanol solution (short: graded ethanol concentration fixation-GECF) was employed in the 48 hours before our experiments, and then, samples were placed in an upright position in a plastic tube to facilitate the synchrotron-based PPCT experimental setup and to avoid motion artifacts associated with deformation or degeneration of the fresh tissues during the measurement with a highly brilliant synchrotron X-ray source.

### Image acquisition system

**Fig 2:**
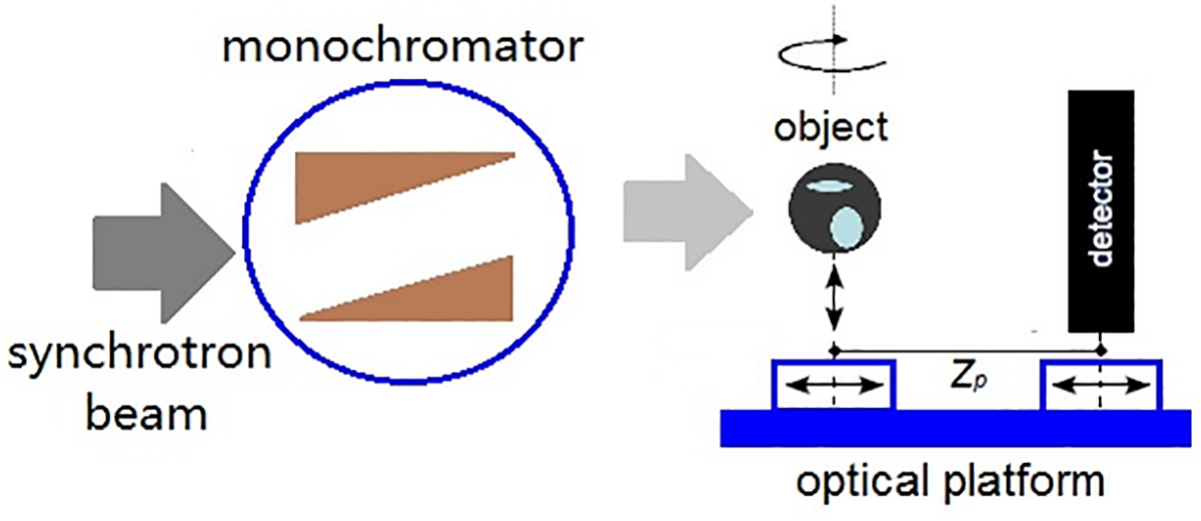
Schematic representation of the PPCTexperimental setup at SSRF.

The experiments were performed at the X-ray imaging and medical application beamline (BL13W1)of Shanghai Synchrotron Radiation Facility (SSRF), depicted in Fig 2. The synchrotron beam, produced from the 3.5GeV storage ring (with ring current I = 250mA, top-up mode), is monochromated with a double crystal monochromator (DCM) Si(111) to select an X-ray energy from 8 to 72keV. The optical stage is located atapproximately 35m downstream of the source, where the acquisition of raw experimental data was implemented with the PPCT technique, which is a free-space propagation method without any optical element between the object and detector. The detector (Hamamatsu, Japan) in our experiments consists of a scientific CMOS camera with a 6.5μm pixel size, coupled with a P43 powder (Gd2O2S: Tb+) phosphor screen with a 10μm thickness for converting the incident hard X-rays into visible light imaging. Propagation over an object-to-detector distance (ODD = *Z*_*p*_), as shown in Fig 2, results in a Fresnel diffraction pattern that contains the contribution from the phase distortion induced by the object. To yield highly detailed three-dimensional views of the non-destructive inner structure of HE rat models based on this PPCT system, a HE sample is rotated around the rotation axis, and a series of raw images are recorded for each projection angle. The tomographic reconstruction can then be achieved from the phase-retrieved projection dataset by using the standard filtered back-projection (FBP) algorithm. The PPCT experimental conditions were as follows: the number of raw imageswas1080 projections over 180° of rotation, acquired at *Z*_*p*_ = 200mmwith aflat-field correction interval of 36°and an exposure time of 500ms per image at 60keV.

### Phase retrieval

The phase retrieval (PR) algorithm based on generalized phase-attenuation duality (PAD)proposed in our previous works [41–43], suitable for meeting the requirement of limited radiation exposure in biomedical imaging, was adopted to recover phase maps induced by specimens from only a single free-space propagation radiograph. The PAD phase retrieval holds in good approximation in our biomedical specimen, as a soft liver tissue is composed mainly of low-Z elements (Z<10) and the hard X-ray energy (≥ 60keV) is high enough to make the Compton scattering dominate the soft tissue attenuation [37, 38]. We can apply a generalized PAD relationship to solve for the phase map *φ*_*θ*_ of a given view angle *θ*, which can be retrieved from a single projection image *I*_*θ*_ in this angle as follows [41, 42]:

$$\varphi_{\theta }\left(\vec{r}\right)=\frac{\lambda r_{e}}{\sigma_{KN}}\cdot \text{ln}\left\{{\left[\text{cos}\left(\frac{\lambda R_{2}}{4\pi M}\nabla^{2}\right)-\left(\frac{2\lambda r_{e}}{\sigma_{KN}}-\frac{\lambda R_{2}}{4\pi M}\nabla^{2}\right)\cdot \text{sin}\left(\frac{\lambda R_{2}}{4\pi M}\nabla^{2}\right)\right]}^{-1}\cdot \left(\frac{M^{2}I_{\theta }\left({\vec{r}}_{D}\right)}{I_{IN}}\right)\right\}$$ (1)

where *λ* is the X-ray wavelength, *r*_*e*_ is the classic atomic radius, and *σ*_*KN*_ is the Klein-Nishina total crosssection for X-ray photon Compton scattering from a single free electron. *M = (R*_*1*_*+R*_*2*_*)/R*_*1*_, where *R*_*1*_ is the source-to-sample distance and *R*_*2*_ is the sample-to-detector distance (*R*_*2*_ = *Z*_*p*_); here, for synchrotron radiation source, *M≈1* because *R*_*1*_*>>R*_*2*_. In addition, ${\left[\text{cos}\left(\frac{\lambda R_{2}}{4\pi M}\nabla^{2}\right)-\left(\frac{2\lambda r_{e}}{\sigma_{KN}}-\frac{\lambda R_{2}}{4\pi M}\nabla^{2}\right)\cdot \text{sin}\left(\frac{\lambda R_{2}}{4\pi M}\nabla^{2}\right)\right]}^{-1}$ is a convolved pseudo-differential operator. Note that the large value of the ratio *λr*_*e*_ / *σ*_*KN*_ reflects the high sensitivity of phase-contrast imaging, which is more than 1000 on the basis of our experimental condition. Prior to the process of phase retrieval for each projection, background correction was performed through the normalization of dark signals and flat-field images.

## Results and discussion

### Calibration of PAD-PPCT with standard specimens

**Fig 3:**
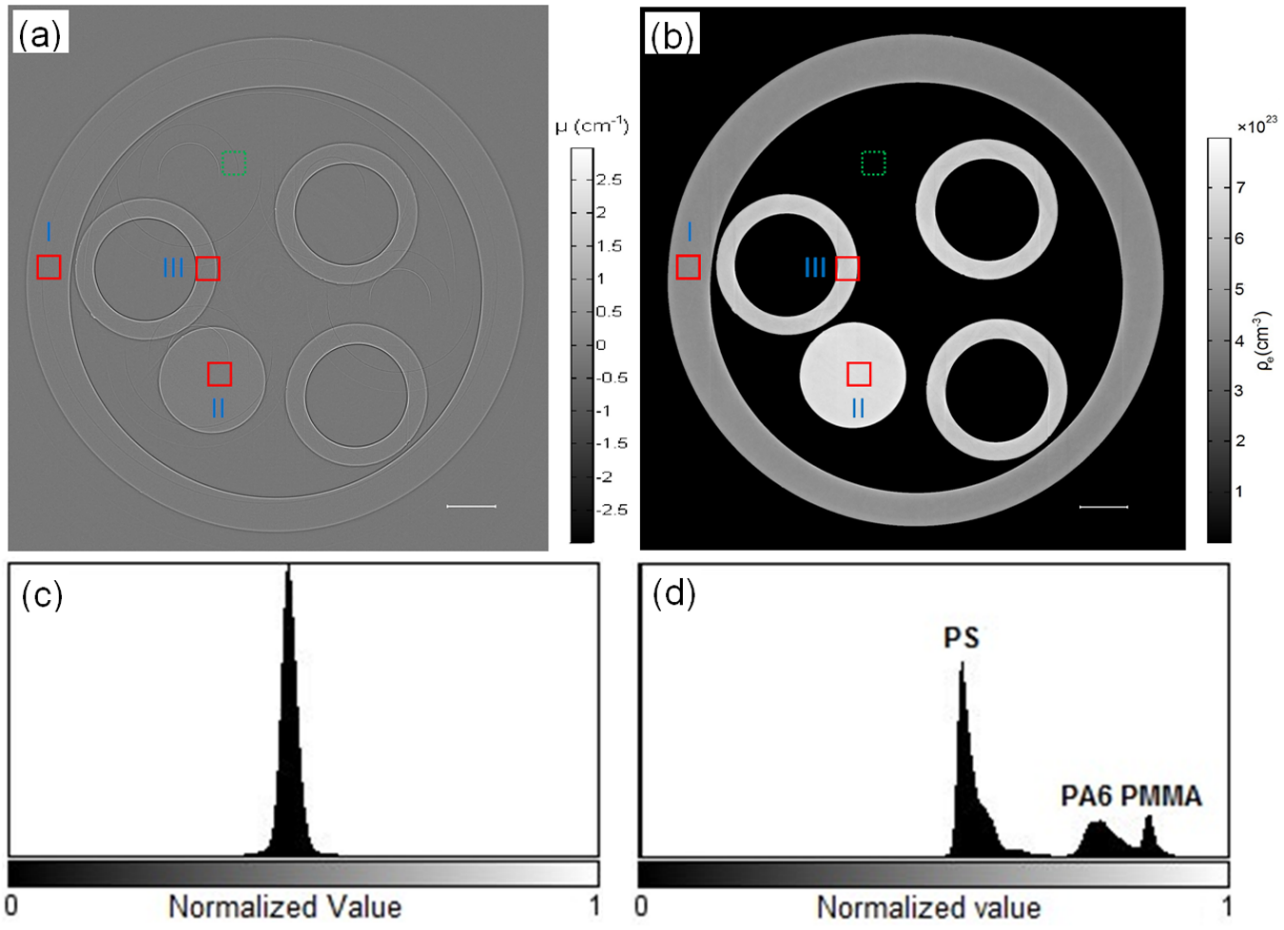
Comparison of reconstructed section imagesof standard material specimens in terms of contrast and histogram. The tomogram (a) and corresponding histogram (c) based on the direct PPCT technique. The tomogram (b) and correspondinghistogram (d) based on the PAD-PPCT technique. The rectangular region of interests (ROIs), depicted with green dashed linesfor background and red solid lines(I/PS, II/PP, III/PMMA) for different materialsamples in (a) and (b), are used for calculation of the contrast-to-noise ratio (CNR). The length of the scale bar is 300μm.

To quantitatively investigate HE specimens, three standard polymer test samples, such as polypropylene (PP, 0.92 g/cm^3^), polystyrene (PS, 1.05 g/cm^3^), and acrylic (PMMA, 1.19 g/cm^3^) tubes, were measured to demonstrate the strikingly enhanced phase contrast of our robust phase retrieval algorithm and calibrate the PAD-PPCT system. Fig 3(a) was directly reconstructed from the PPCT technique without the PAD algorithm and shows strong edge-enhanced material boundaries as a result of the refraction and diffraction of the phase-shifted X-rays. The reconstructed abnormal linear attenuation coefficients at interfaces and boundaries enable the outlines of the three standard materials to be seen clearly, but their bulk attenuation contrasts are very weak because of the weakly absorbing materials. This results in complete overlapping of the three different materials' peaks on a histogram, displayed in Fig 3(c), which is not in accordance with their actual material density distribution. However, the reconstructed section image based on our PAD-PPCT technique, shown in Fig 3(b), exhibited good conformity on both the real material structure and density distributions of the standard specimens, with totally separated material peaks in Fig 3(d). The comparison of both experimental results demonstrated that the PAD-PPCT technique has great potential for discriminating and visualizing the tiny density and structural differences of low-Z materials or soft biological tissues.

**Fig 4:**
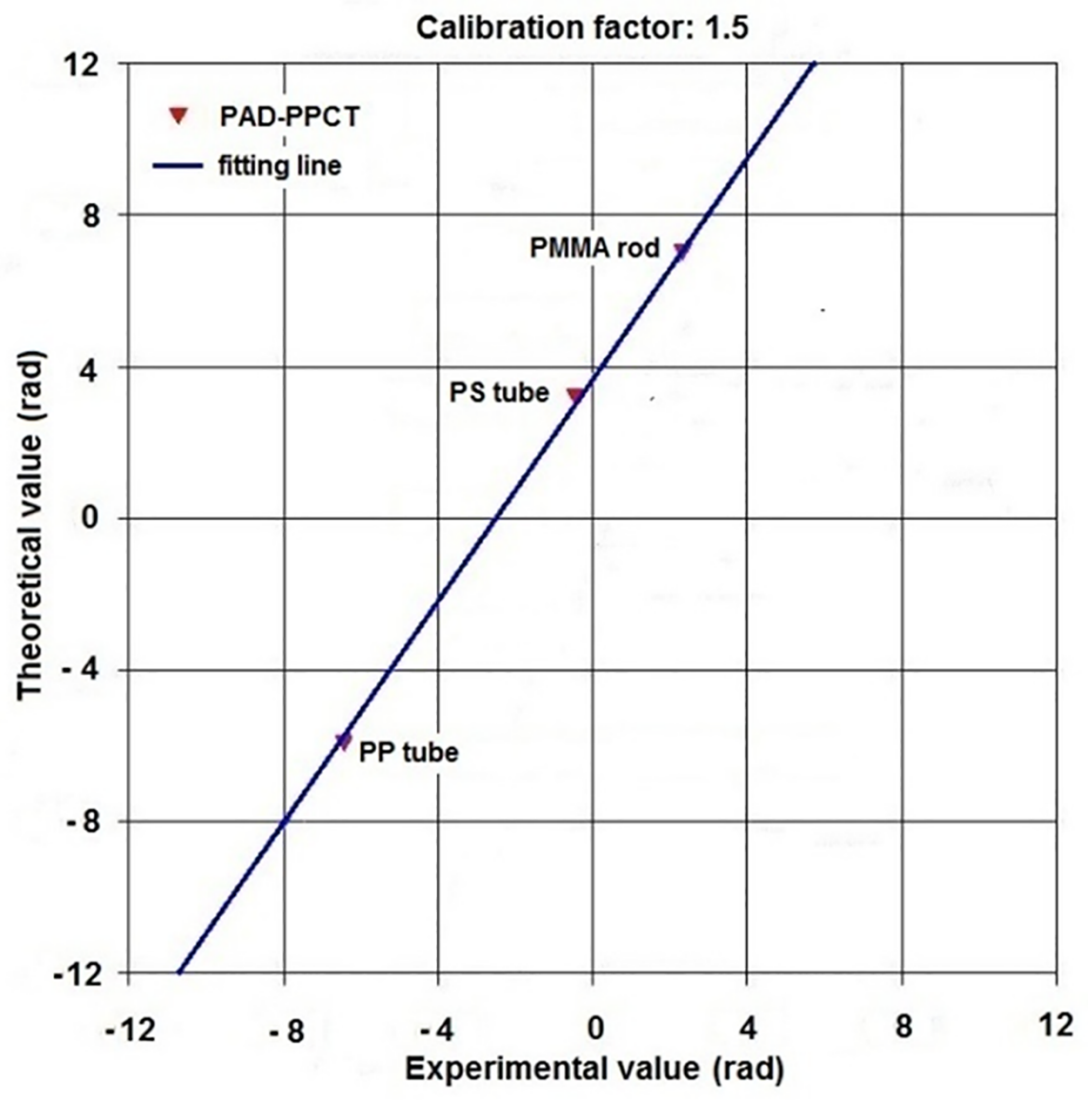
Relationship between the theoretical phase-shift values and the measuredvalues based on PAD-PPCT. The calibration factor of our imaging system is 1.5.

For quantitative analysis of experimental results and estimation of the HE specimen's density distributions, it is necessary to calibrate this imaging system through the measured pixel values obtained from the PAD-PPCT experiments, which were compared to their theoretical phase shift values based on the fitting line by using the least square method. The calibration result is described in Fig 4, and a linear relationship was found between the experimental and theoretical values for the quantitative measurement. The calibration factor of the PAD-PPCT system is 1.5, mainly depending on the X-ray detector.

### Measurement of HE specimens based on PAD-PPCT

**Fig 5:**
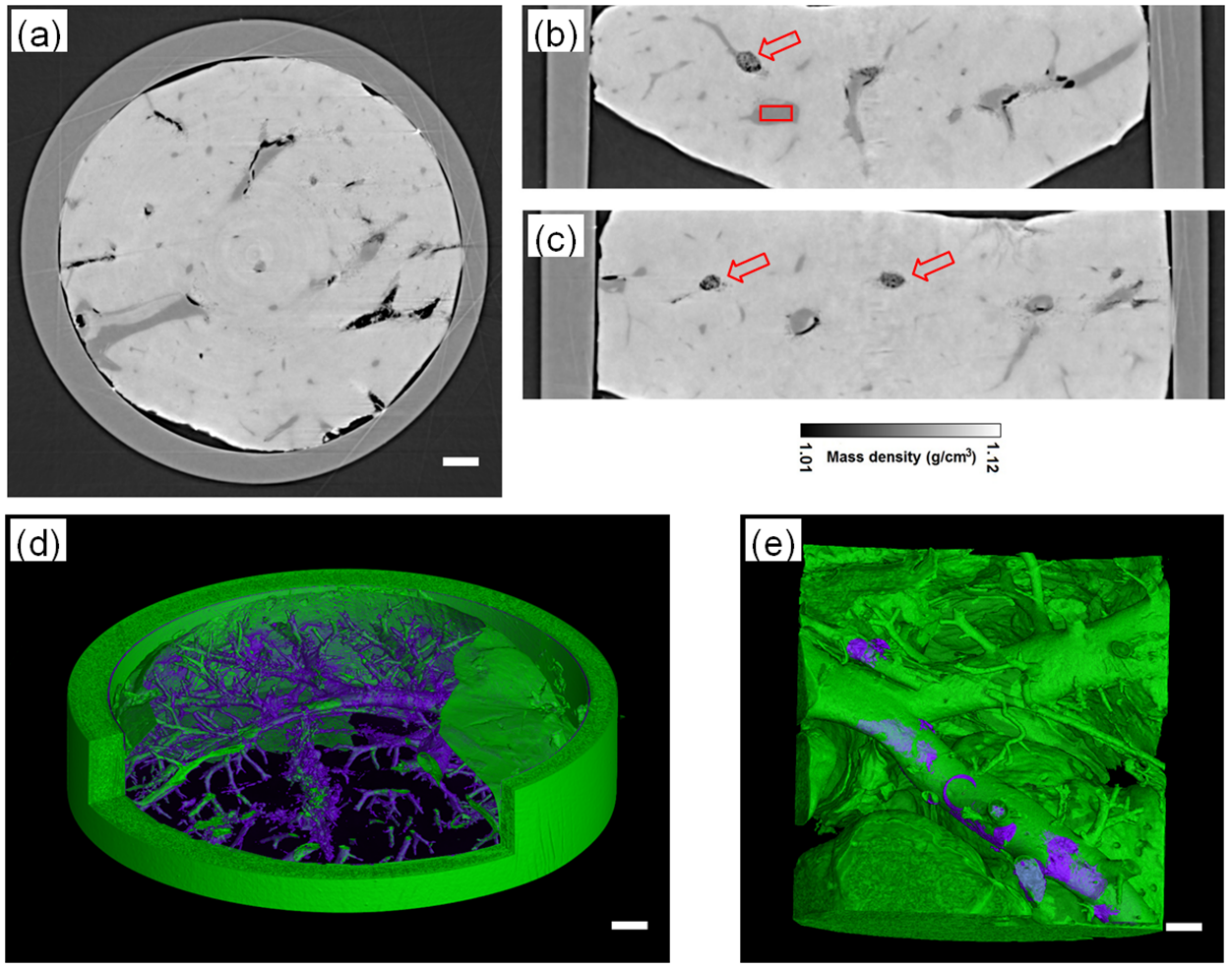
Reconstructed results ofthe early HE specimen of the two-week rat model based on PAD-PPCT. (a), (b)and (c) are,respectively,transversal, sagittal and coronalsection imagesdisplayed on a mass density gray scale. The red arrows in (b) and (c) indicate the early HE lesions. (d) and (e) are the 3D renderings of entire volumetric early HE lesions of different HE specimens in the two-week group with different length scales. The length scalebars are, respectively, 200μm and 100μm in (a)-(d) and (e).

**Fig 6:**
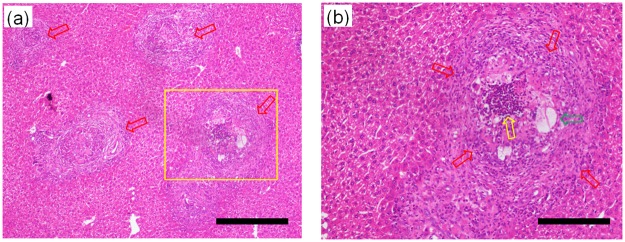
Histopathological examinations of the early HE specimens of the two-week rat modelwith thehematoxylin and eosin (H&E) staining process, corresponding to the light dark area indicated by the red rectangle in Fig 5(b), using 10× and 40× objective lens. (b) corresponds to the yellow rectangular area in (a). The length scalebars are, respectively,20μm and 5μm in (a) and (b).

Without using a contrast agent or staining methods, the phase contrast sectional images of a two-week HE rat model are displayed in Fig 5(a), 5(b) and 5(c), respectively representing transversal, sagittal and coronal slices reconstructed only by the PAD-PPCT technique. It can be obviously seen that the experimental results show clear contrast between the HE lesions and periphrastic normal tissues with the gray scale of mass density, such as the early tiny HE cystic / necrosis lesions due to alveolar *Echinococcus multilocularis* infection indicated by the red arrows in Fig 5(b) and 5(c). However, the lesions cannot be found by MRI examination, and such a small change could not be clearly visualized by conventional absorption contrast imaging. These small changes in the density of tissues were depicted as changes in the apparent image contrast via the presented phase contrast imaging technique and the three-dimensional renderings of the HE specimen, shown in purple color in Fig 5(d) and 5(e) with different scale levels. This technique clearly revealed the entire volumetric endovascular HE lesion microstructure and distribution at an early stage due to the advantage of density resolution as depicted in Fig 3, demonstrating that the early tumor-like HE disease propagates along the microvascular blood networks. With careful visual inspection, there are some light dark areas, such as those indicated with a red rectangle in Fig 5(b), and they were examined by H&E staining microscopy for further verification, shown in Fig 6(a) and 6(b). It can be obviously found that there are some bright HE immune response rings, as indicated by red arrows in Fig 6(a). The enlarged inset of Fig 6(b), corresponding to the yellow rectangle in Fig 6(a), shows the early hepatic echinococcosis and the triggered cluster of microvesicles smaller than 2μmsurrounded by macrophages, epithelioid cells and lymphocyte infiltration.

**Fig 7:**
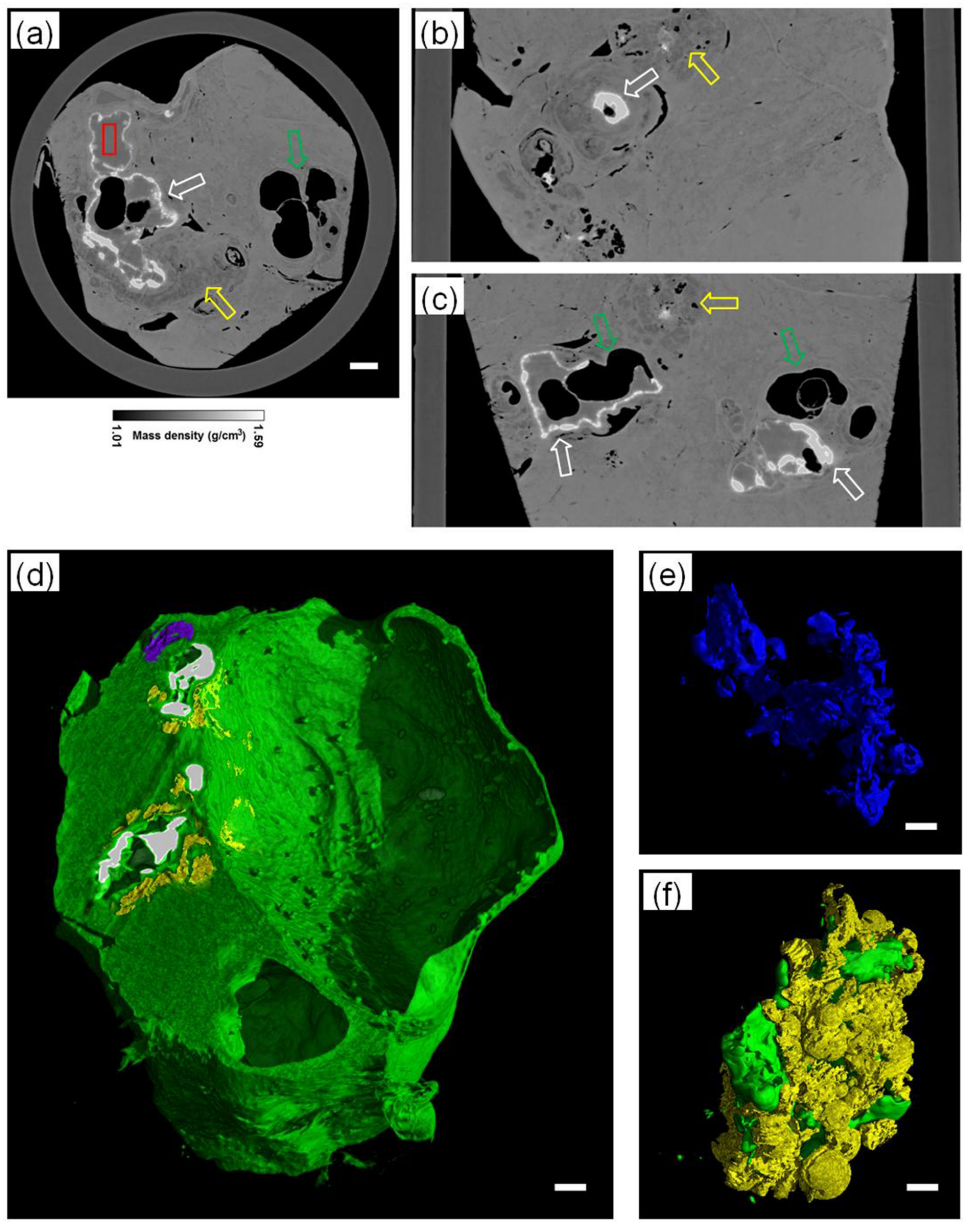
Reconstructed results ofthe developed HE specimen of the two-monthrat model based on PAD-PPCT. (a), (b)and (c) are,respectively,transversal, sagittal and coronal section imagesdisplayed on a mass density gray scale. The green arrows denote HE-induced cysts, white arrows denote micro-calcifications, yellow arrows denote inflammation areas, and red rectangle corresponds to H&E examination. (d) is the 3D rendering of entire volumetric developed HE lesions, and (e) and (f) are the quantitative segmentation resultsof (d). The length scalebars are, respectively, 200μm and 100μm in (a)-(d) and (e)-(f).

**Fig 8:**
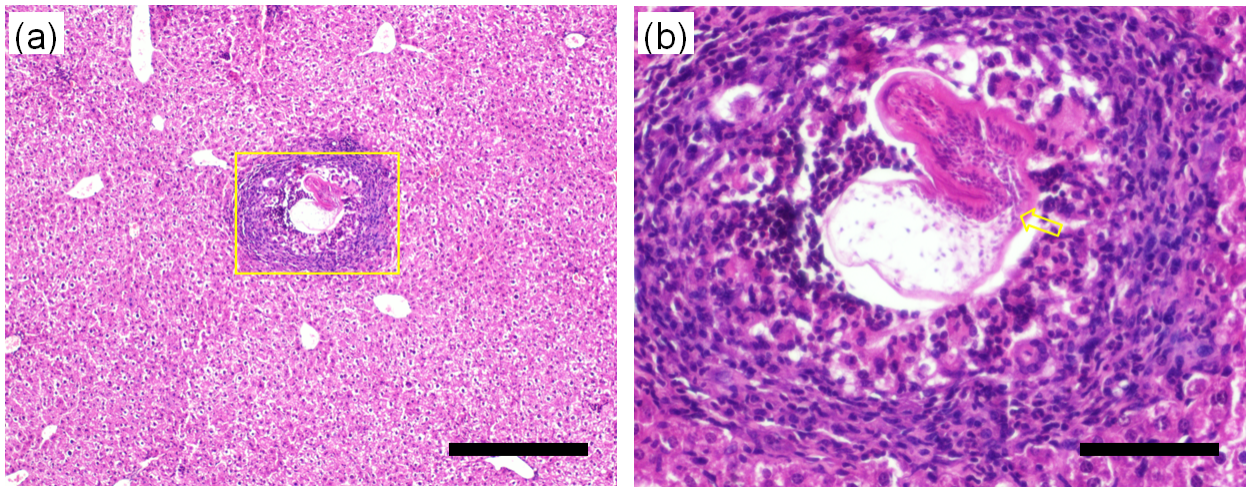
Histopathological examinations of the early HE specimen of the two-monthrat model with thehematoxylin and eosin (H&E) staining process, corresponding to the area indicated by the red rectangle in Fig 7(a), using 10× and 40× objective lens. (b) corresponds to the yellow rectangle area in (a). The length scalebars are, respectively,20μm and 5μm in (a) and (b).

Another experiment was performed with an HE specimen selected from the two-month rat model group to investigate the micro-morphological characteristics of developed HE based on our PAD-PPCT technique. Fig 7(a), 7(b) and 7(c) are reconstructed sectional images through the different orientations displayed on a mass density gray scale, showing the diverse HE lesions, such as microcysts (green arrows), micro-calcifications (white arrows), and inflammation areas (yellow arrows). Moreover, the 3D rendering of Fig 7(d) enables the visualization of different HE lesions with different colors vividly, such as micro-calcifications (white), fibrosis tissues (yellow), HE parenchyma (blue), *E*.*multilocularis* metacestodes and entire HE micro-cavities (green holes). Due to the high density sensitivity and striking contrast-to-noise ratio of our PAD-PPCT technique, 3Drenderings of quantitative segmentation, as shown in Fig 7(e) and 7(f), exhibited the volumetric distribution and structural characteristics of different HE lesions with small density differences, such as HE parenchyma rendering (e) and inflammation area rendering (f). In addition, Fig 8(a) and its enlarged inset (b) indicated with the yellow rectangle in Fig 8(a) show the results of histopathological examination using H&E staining of the HE-induced inflammation area, corresponding to the red rectangle in Fig 7(a). This figure clearly demonstrated that the developed cysts induced by hepatic echinococcosis triggered a strong HE immune response, agreeing with the inflammation areas in Fig 7(a)–7(c).

### Analysis of contrast-to-noise ratio (CNR)

To quantitatively analyze the quality of the tomographic images obtained from the PAD-PPCT technique, we examined the density resolution (in view of the contrast-to-noise ratio: CNR). For the calculation of CNR based on these selected pairs of ROIs, as seen in Fig 3(a) and 3(b), the following formula was used:

$$CNR=\frac{\left|S_{obj}-S_{bg}\right|}{\sqrt{\sigma_{obj}^{2}+\sigma_{bg}^{2}}}$$ (2)

where *S* represents the average of a homogeneous material, and *σ* represents its standard deviation. The manually defined object (red solid rectangles: I/PP, II/PS, III/PMMA) and background region (green dashed rectangle), indicated in Fig 3(a) and 3(b), are denoted with the subscripts *obj* and *bg*, respectively. The calculated results are the following: CNR_PPCT_I, II, and III equal to 4.35, 5.21, and 5.98 and CNR_PAD-PPCT_I, II, and III equal to45.61, 55.36, and 61.23, respectively. These results indicated that the bulk CNRs with the PAD-PPCT technique were more than 10 times higher than those with the direct PPCT method. This significant enhancement in the bulk CNR implies that the PAD-PPCT method has great potential for suppressing noise, enhancing image contrast, and reducing the radiation dose. Actually, our experimental data demonstrated that the PAD-PPCT method plays an important role in quantitative measurement and segmentation of HE specimens in this disease research.

### Estimation of tissue's mass density

For the PAD-PPCT method, the reconstructed results generally describe the distribution of the object’s electron density, in which a pixel value denotes a specimen’s refraction index decrement *δ*. To obtain the mass density distribution of our HE specimens, the relationship of both mass density *ρ* and refraction index decrement *δ* can be calculated from the formula below:

$$\delta =\frac{\lambda^{2}r_{e}}{2\pi }\cdot N_{a}\cdot \rho \cdot \sum w_{i}\frac{Z_{i}}{A_{i}}$$ (3)

where *λ*, *r*_*e*_, and *N*_*A*_, respectively, represent the X-ray wavelength, the classical electron radius (2.82×10^-15^m), and Avogadro’s constant (6.022×10^23^), *w*_*i*_ is the weight fraction of the *i-th* element of a molecule, and *Z*_*i*_ and *A*_*i*_ are the atomic number and atomic weight of the corresponding *i-th* element, respectively. For our estimation, the ratio value of *Z*_*i*_*/A*_*i*_ was assumed as 0.55; based on the average for soft tissues (referring to the NIST database),∑*w*_*i*_ is actually equal to 1. According to the Eq (3), the density variance Δ*ρ* is expediently estimated from the reconstructed Δ*δ* in X-ray PAD-PPCT. A measured Δ*δ* value can be calibrated using standard specimens under a given experimental condition. Our experimentally measured Δ*δ* values were multiplied by the calibration factor 1.5. Thus, the mass density distributions of the specimens from both HE rat model groups are displayed with mass density scalebars in Figs 5 and 7.

### Quantitative segmentation and statistical analysis

Without the staining process and micron-level slice cutting of our experimental HE specimens, the quantitative pathological measurement of HE specimens based on the single-distance synchrotron PAD-PPCT technique with a simple experimental setup was investigated and demonstrated the potential of our presented method, especially for the quantitative segmentation of different types of HE lesions due to the striking CNR advantage and high density resolution of the PAD-PPCT method, as shown in Fig 7 (e) and 7(f). Therefore, it is feasible to obtain improved image contrast and higher spatial resolution to extract a type of HE lesions with tiny density changes from weak-absorption biomedical soft samples by using the robust phase retrieval based segmentation, which benefits visualization and quantitative measurement of inner pathological structures and lesion characterization in HE tissues. For example, it can visually supply are liable morphological indicator of disease activity, and its absence could indicate degeneration, revealing the parasitic metabolic activity at the early stage of pathology.

**Fig 9:**
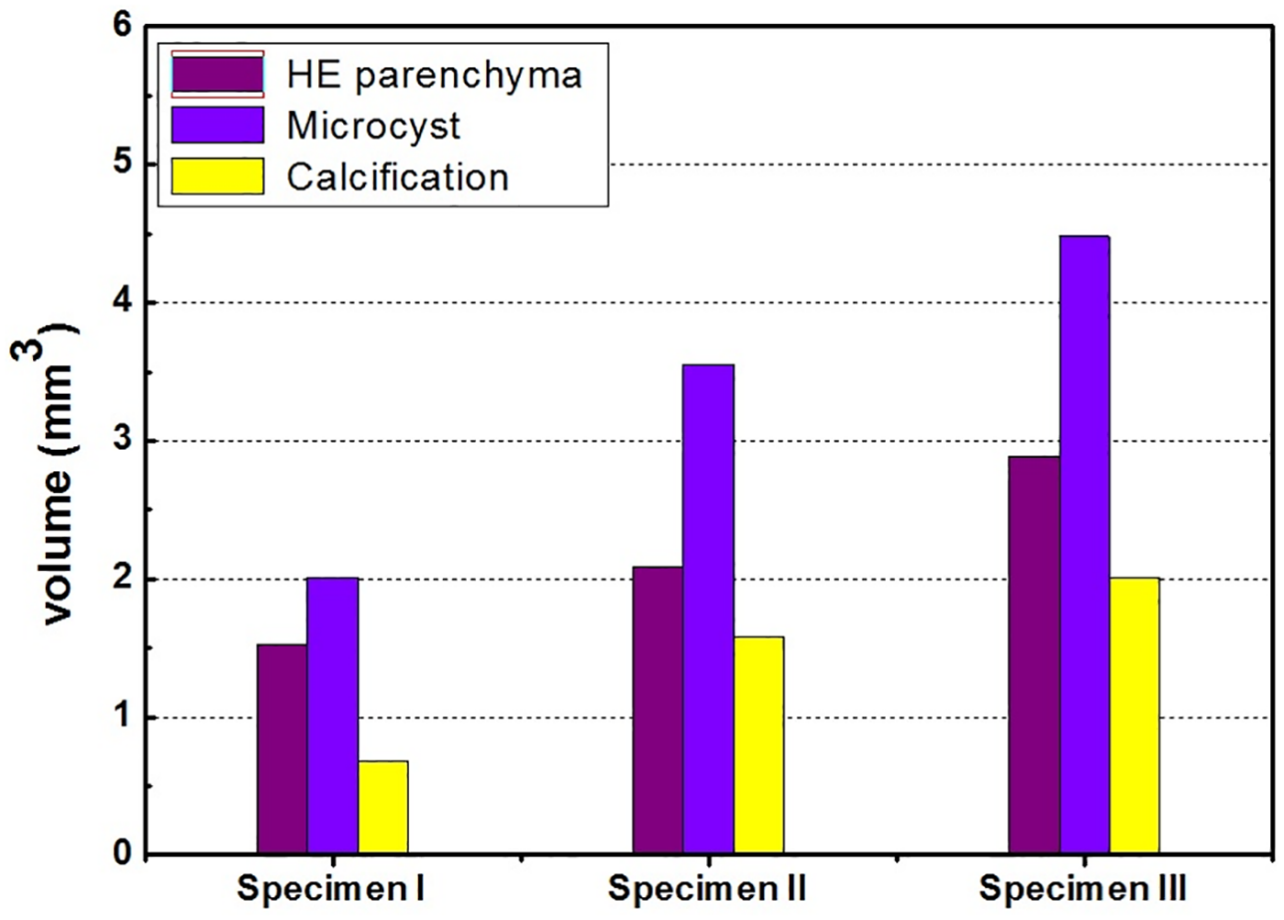
Statistical volumetric results of three types of HE lesions based on quantitative PAD-PPCT segmentation.

In addition, the statistical analysis of different HE lesion volumes based on the effective 3D segmentation can be obtained and is presented for three HE rat disease models (specimens I, II, and III), as shown in Fig 9, providing the quantitative estimations of the damage level of different types of HE lesions.

## Conclusion

The PAD-PPCT imaging technique showed excellent density sensitivity and spatial resolution in 3D visualization of hepatic *echinococcosis* (HE) pathology based on our experimental results with rat HE disease models, effectively displaying enhanced phase contrast of early HE lesions of blood vascular invasion and different types of micro-morphological characteristics of HE lesions in mature rat models, which will play an important role in the pathological examination and assessment of therapeutic effects. Generally, it is helpful to quantitatively extract and analyze the morphological characteristics of soft tissues or organs closely related to the occurrence and development of HE or other diseases.
